# The Importance of Maintaining and Improving a Healthy Gut Microbiota in Athletes as a Preventive Strategy to Improve Heat Tolerance and Acclimatization

**DOI:** 10.3390/microorganisms12061160

**Published:** 2024-06-06

**Authors:** Sergi Cinca-Morros, Jesús Álvarez-Herms

**Affiliations:** 1 Microfluidics Cluster UPV/EHU, Analytical Microsystems & Materials for Lab-on-a-Chip (AMMa-LOAC) Group, Analytical Chemistry Department, University of the Basque Country UPV/EHU, 01006 Vitoria-Gasteiz, Spain; 2 Microfluidics Cluster UPV/EHU, BIOMICs Microfluidics Group, Lascaray Research Center, University of the Basque Country UPV/EHU, 01006 Vitoria-Gasteiz, Spain; 3 Physiology and Molecular Laboratory (Phymolab), 40170 Collado Hermoso, Spain; jesusah80@gmail.com

**Keywords:** acclimation/acclimatization, heat stress, gut microbiota, intestinal dysbiosis, intestinal permeability, athletes

## Abstract

Exposure to passive heat (acclimation) and exercise under hot conditions (acclimatization), known as heat acclimation (HA), are methods that athletes include in their routines to promote faster recovery and enhance physiological adaptations and performance under hot conditions. Despite the potential positive effects of HA on health and physical performance in the heat, these stimuli can negatively affect gut health, impairing its functionality and contributing to gut dysbiosis. Blood redistribution to active muscles and peripheral vascularization exist during exercise and HA stimulus, promoting intestinal ischemia. Gastrointestinal ischemia can impair intestinal permeability and aggravate systemic endotoxemia in athletes during exercise. Systemic endotoxemia elevates the immune system as an inflammatory responses in athletes, impairing their adaptive capacity to exercise and their HA tolerance. Better gut microbiota health could benefit exercise performance and heat tolerance in athletes. This article suggests that: (1) the intestinal modifications induced by heat stress (HS), leading to dysbiosis and altered intestinal permeability in athletes, can decrease health, and (2) a previously acquired microbial dysbiosis and/or leaky gut condition in the athlete can negatively exacerbate the systemic effects of HA. Maintaining or improving the healthy gut microbiota in athletes can positively regulate the intestinal permeability, reduce endotoxemic levels, and control the systemic inflammatory response. In conclusion, strategies based on positive daily habits (nutrition, probiotics, hydration, chronoregulation, etc.) and preventing microbial dysbiosis can minimize the potentially undesired effects of applying HA, favoring thermotolerance and performance enhancement in athletes.

## 1. Introduction

Over the last decades, athletes have starting to include different strategies of passive and active exercise in hot conditions (passive heat) to promote recovery and thermotolerance in subsequent exercise in the heat [1,2,3,4]. Exposure to heat promotes adaptive responses of acclimation or acclimatization, depending on whether the exposure occurs in artificial (acclimation) or natural (acclimatization) warm environments [1]. Heat acclimation/acclimatization, hereafter HA, is used among athletes to protect health and improve performance in hot environments [1]. Long-term HA strategies, which include regular passive or active heat exposure for more than 10 days, achieve the best results compared to short-term interventions [4]. To date, there have been a large number of protocols and guidelines described for long-term HA procedures [3,4,5,6,7]. During exercise in the heat, premature exhaustion and shock due to heat stress (HS) can be common in athletes not acclimatized to heat and are the result of a complex combination of heat cytotoxicity (including endotoxemia from the gut), impaired coagulation, and systemic inflammatory response syndrome (SIRS), which is followed by deterioration of organ responses and ultimately, reduced physical performance. The prevention of heat stress is now considered to be more beneficial than treating the condition. Therefore, providing strategies that promote the prevention of premature heat fatigue would be of interest for athletes, coaches, and scientist.

Biologically, prolonged and intense exercise elevates body temperature, promoting blood redistribution to active muscles, which reduces intestinal blood flow by 80% [8]. This effect promotes intestinal ischemia that exacerbates damage to the epithelial cells and tight junction proteins making up the intestinal mucosa’s physical barrier [9]. The gastrointestinal (GI) tract exhibits key functions in maintaining whole-body homeostasis and health. The GI tract is the organ directly responsible for absorbing nutrients, and it acts as a physical and immunological barrier against the entry of noxious compounds into the interior milieu [10]. Impairment of the gut barrier integrity is often involved in various GI and extra-GI diseases, resulting in reduced physical performance. On the other hand, the gut microbiota (GM) colonize the GI tract and develop multiple systemic functions for the host at digestive [11,12], metabolic [11,12], immunological [11,12,13], homeostatic [11,12,14,15,16], and structural [11,12] levels. In fact, the GM is directly involved in maintaining the epithelial barrier integrity through bacterial activity, producing and synthesizing mucus and short-chain fatty acids (SCFAs) [11,12,17]. Thus, the GM interacts directly with the immune and neural systems through metabolites and nerves (gut–brain axis), modulating the inflammatory response, and preventing harm to the intestinal barrier [13]. Furthermore, the GM contributes to maintaining the appropriate intestinal pH, temperature, and oxygen levels for different structures of the intestine [11,12,14,15,16]. Maintaining a healthy gut microbiota can be achieved by maintaining a higher diversity, stability, and balance of the bacterial ecosystem [18]. For athletes, maintaining a healthy GM is key to promoting adaptive benefits that prevent aberrant responses such as those occurring during a state of gut dysbiosis, characterized by local inflammation, leaky gut syndrome, and impaired metabolic and endocrine functions [19]. In this regard, the therapeutic role of maintaining a healthy gut microbiota (GM) has also been reported in the prevention of heat stroke through the deterrence of systemic endotoxemia [20]. 

Conversely, the imbalance of the GM is defined and described in the literature as a dysbiosis that contributes to a worsening of the symbiotic functions in the host [17]. Such a condition can be reached following exposure to different stressful stimuli and negatively result from a life style that includes poor nutrition, exposure to toxics and pollution, chronodisruption, certain acquired pathologies, and sedentarism [21]. An aberrant consequence of gut dysbiosis is leaky gut syndrome, in which intestinal permeability is impaired, resulting in the higher translocation of bacteria and toxins from the gut into the bloodstream (endotoxemia) [22,23,24]. Systemic endotoxemia results in a systemic inflammatory and immune response that directly impairs health and physical performance [25]. Thus, the chronicity of systemic inflammation and immune overreaction can lead to a negative physiological spiral, compromising both the health and physical performance of athletes [26]. The pathophysiology occurring during heat exposure can be aggravated when athletes suffer from a state of GM dysbiosis. In this regard, Costa et al. [27] describe how improving the balance of the gut microbiota (GM) in athletes, including epithelial barrier health, could improve the systemic state during exercise in the heat, decreasing the inflammatory response and endotoxemia induced by the gut. In rats, Cao et al. [28] demonstrated that improving the gut microbiome balance and its functions in regards to gut barrier integrity (mucus, cell integrity, and selective permeability) induced protective properties under heat stress. The mucus layer acts as an important mechanism for protecting the host against microbial invaders and contributes to the mutualism between the host and the microbes [29]. The loss of mucus layer thickness would be a consequence, as well as a precursor for both negative as maladaptive responses to HA and other physically elevated demands in athletes. 

Therefore, athletes must take into consideration their own systemic health [30], including their GM balance and the integrity of their GI functions prior to including highly specific training methods, such as HA. Future studies require the investigation of direct strategies that prevent and treat GI complications produced by extreme environmental conditions such as heat [31] or altitude [32].

Here, we investigate the direct consequences induced by a state of GM dysbiosis on maladaptive responses suffered by athletes exposed to HS. The main goal of the present study is to highlight the importance of maintaining a healthy GM balance in athletes as a therapeutic treatment, which can specifically contribute to the prevention of premature fatigue during HS exposure and exercise.

## 2. Heat Stress Stimulus and Gut Dysbiosis in Athletes

The physical preparation of high-performance athletes is very difficult and includes repeated highly specific and intense stimulus (elevated consumption of simple carbohydrates [30] and proteins [33], hypoxic exposure and training [34], and heat exposure and training [4]). Extensive evidence has shown that heat exposure (active and passive) can promote health and physical performance benefits [3,32,33,34]; however, the specific threshold that negatively affects health has not been completely explored. Until recently, most research on this topic has focused on how heat stress promotes intestinal alterations, such as burns, in patients with heat-related pathologies [35,36,37]. However, these specific interventions require efficient systemic responses to maintain and/or improve physiological performance under such stress. The maintenance of the systemic health of athletes is the main factor for obtaining the benefits of these specific interventions [30]. For example, in a systemic context, where athletes experience a decline in health due to chronic stress, higher levels of immune response and inflammation increase the allostatic load and can induce more physiological damage, impairing physical performance. It is important, therefore, to understand the mechanisms that improve adaptive responses in both the short and long term, including a healthy GM and its communication with the athlete’s organisms.

The increased plasma endotoxemia from leaky gut syndrome is suspected to be an important etiological factor in the circulatory shock that accompanies premature HS intolerance in humans [38]. The exposure to HS and maximal exercise aggravates endotoxemia in the context of impaired gut permeability, possibly due to the reduction of hepatic detoxification. During HA and exercise, the reduction of portal vein blood flow, combined with thermally altered hepatocyte function, severely reduces the capacity to detoxify a surge of endotoxin [39].

**Figure 1:**
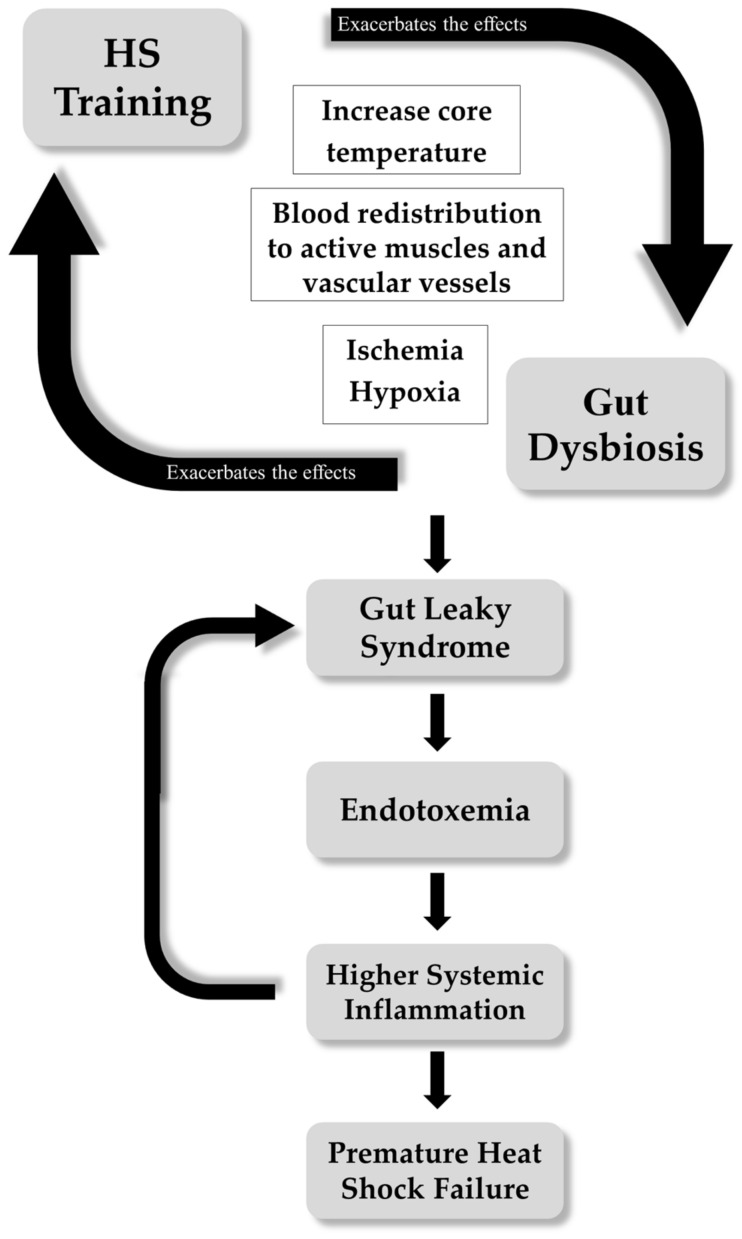
Heat stress (HS) training and exposure promote ischemia in the intestine through the blood redistribution to active muscles and vascular vessels. The main goal of the body during HS is maintain homeostatic temperature. Gut dysbiosis is a negative health condition for the body, impairing symbiotic functions of the microbiota and aggravating intestinal dysfunctions, presenting as impaired gut permeability. Gut permeability dysfunction allows for the passage of endotoxins into the blood, increasing the systemic inflammatory response and reducing the time to exhaustion during exercise in a hot environment.

The non-adaptive effects of HS on performance and health have been related to exacerbated cardiovascular, metabolic, and hormonal responses [7]. At the gut level, HS can lead to leaky gut syndrome, which is associated with intestinal ischemia. During HA and exercise, a systemic blood flow redistribution from splanchnic and renal vascular beds to the muscular and cutaneous vascular beds exists, ensuring an adequate energy and oxygen supply to the active muscles and facilitating heat dissipation [40]. However, gut ischemia and its consequent hypoxia increases metabolic stress [41], which directly alters the structure of the enterocyte membrane [22], involving tight junctions (TJs), which become damaged, allowing for the translocation of lipopolysaccharides (LPS) from Gram-negative bacteria (mainly proteobacteria) into the circulation [22]. Gram-negative bacteria found in the small and large intestine are harmful to the body due to an endotoxin unit located in the bacterial outer membrane [23]. This translocation of LPS from the luminal side to the bloodstream stimulates a systemic immune response characterized by the release of proinflammatory cytokines IL-6 and TNF-α [42]. It also activates innate immune production (circulating monocytes and tissue macrophages like Kupffer cells) of anti-inflammatory cytokines IL-1 [43]. This inflammatory response further affects tight junctions, which show increased leakage of bacterial LPS on the basolateral side [44]. Along with the cytotoxic effects of hyperthermia produced by HS and exercise itself, dehydration often exacerbates these effects, compromising the thermoregulatory capacity [45]. This situation further aggravates permeability, and the blood LPS concentration reaches a threshold that can trigger a systemic inflammatory response (SIR) (see Figure 1) [39].

Previous studies have shown that athletes who maintain a healthy gut homeostasis can perform physical activity free of adverse heat-related consequences, tolerating higher core temperatures (Tc), even up to 42°C [46,47,48,49]. In this context, research studies have proposed different preventive measures, focusing on minimizing the systemic effects of increased body temperature [50,51,52]. However, no previous studies have suggested that athletes who improve their GM health could preserve their intestinal functions and reduce the premature heat-shock and/or systemic failure induce by exacerbated endotoxemia and inflammation derived from the gut. These preventive factors related to gut health may play a crucial role in the pathophysiology of HS beyond the effects of heat stress, as suggested by previous research [26,53,54]. In this regard, Armstrong et al. [39] proposed that higher tolerance to moderate levels of LPS produced during HS and maximal exercise could be a plausible explanation for the superior tolerance to endotoxemia in athletes acclimatized to hyperthermia.

**Figure 2:**
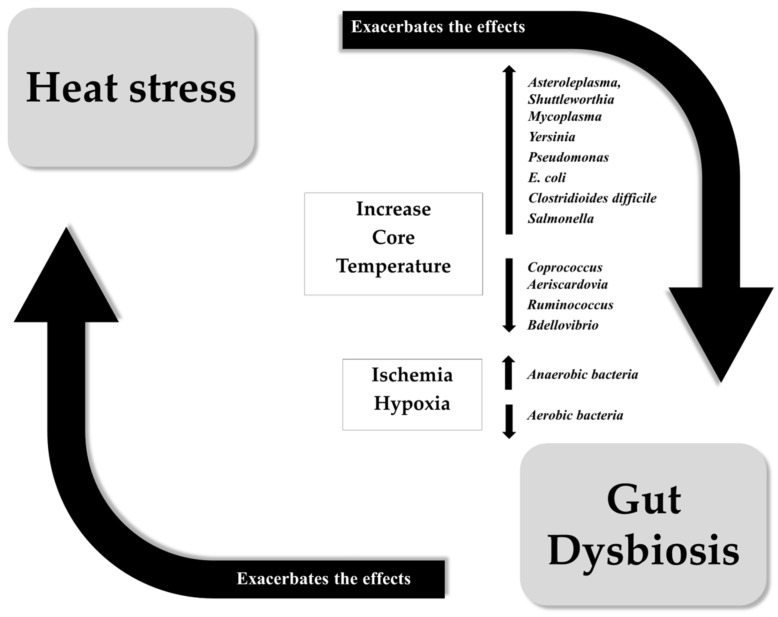
Heat stress can lead to gut dysbiosis through the elevation of certain thermotolerant bacteria, while reducing other beneficial bacteria. The reduction of blood perfusion during exercise produces ischemia hypoxia in the gut, possibly increasing the GM dysbiosis and exacerbating the effects of heat stress during exercise.

In the short-term, acute HS appears not to affect the diversity and abundance of protective commensal bacteria (those more favorable to host gut health, such as *Lactobacillus*, *Bifidobacterium*, *Streptococcus*, *Akkermansia muciniphyla*, *Faecalibacterium prausnitzii*, *Roseburia*, or *Eubacterium*) [55,56,57,58]. However, applying chronic passive and active heat during HA stimuli in athletes may aggravate leaky gut syndrome and potentiate systemic endotoxemia. This could trigger systemic inflammation similar to that observed in pathologies associated with heat stress and significantly impair physical performance (see Figure 1). Intestinal microorganisms are sensitive to changes in systemic temperature. The increase in internal temperature derived from exercise in HA could enhance the emergence of potential pathogens that would directly exacerbate dysbiosis, intestinal leaky gut syndrome, and consequently, systemic endotoxemia and inflammation (see Figure 2). It has been reported that each bacterium require a specific environmental milieu to live and growth adequately. In this regard, some pathogenic bacteria, such as *Escherichia coli* and other members of the *Enterobacteriaceae* family, are thermotolerant, able to survive at temperatures both cooler and warmer than those of the typical endothermic host. *Yersinia enteropathogenic* can grow at temperatures close to 0 °C [59], while laboratory strains of *Escherichia coli* can grow between approximately 8 °C [60] and 42 °C, and adapt to grow at temperatures of 48 °C or higher [61]. Members of the *Proteobacteria* phylum are considered functionally flexible in response to various environmental stresses. Specifically, pathogens like *Salmonella*, *Yersinia*, *Pseudomonas*, and pathogenic *Escherichia coli* use host temperature as an environmental cue to upregulate the virulence genes [62,63,64]. These genes respond more strongly to fever-like temperatures, such as 42 °C, than to temperatures below 37 °C [64], and an enzyme in *Pseudomonas aeruginosa* shows increased efficiency up to 45 °C [64]. *Clostridioides difficile*, another relevant intestinal pathogen, grows equally well at 37 °C and 41 °C in vitro [65,66]. In a study using growing pigs subjected to chronic heat stress, intestinal dysbiosis and increased permeability were observed, characterized by an increase in the presence of potential pathogens like *Asteroleplasma*, *Shuttleworthia*, and *Mycoplasma*. Additionally, a suppression of beneficial bacteria species such as *Coprococcus* and *Aeriscardovia*, which are associated with intestinal immune function, was observed [67].

## 3. Could Gut Health Improve Heat Stress Tolerance in Athletes?

The use of biomarkers to monitor HS response (molecular, physiological, and behavioral) can be a valuable tool for assessing an athlete’s resilience under such conditions. To maintain homeostasis during HS, athletes fight to adjust their physiological and behavioral responses to positively adapt. The regular stimulus of heat exposure in athletes possibly alters the behavioral responses, including food and hydration intake, the frequency and duration of sleep, and normal activities, including resting. From a molecular perspective, biomarkers such as heat shock protein 60 [39] or interleukins [35] are being used to monitor the response to HS. Thus, GM analysis could also be used as a biomarker contributing to an early diagnosis of HS intolerance by detecting GM dysbiosis. [68]. 

The gut is one of the major target organs affected by specific interventions caused by exercise, altitude, nutrition, and heat stress. High-intensity exercise and HA promotes damage of the mucosal epithelia, increasing immune activity and leaky gut syndrome. Moreover, HS promotes changes in regards to GM diversity [69]. Recent studies have suggested that heat acclimatization improves physiological performance during HS through systemic responses, but this may also be related to GM adaptation [55]. It has been shown that after HA, a significant decrease in potentially pathogenic bacteria occurs, whereas beneficial bacteria increase significantly [9,55,70]. Therefore, optimizing beneficial bacterial phenotypes from the GM (for example, higher levels of bacterial precursors of efficient gut permeability, short-chain fatty acid production, secondary bile acid production) confers potential benefits for responding and adapting to HS and physical exercise. Interventions focused on precise nutrition and probiotic intake [55,71] could be necessary when athletes include heat exposure during their training and competitive routines.

### 3.1. Specific Nutrition and Hydration Balance to Improve Gut Health and HA Tolerance in Athletes

The loss of bacterial homeostasis in the gut (dysbiosis), with the presence of impaired epithelial permeability (leaky gut syndrome), is a catastrophic condition for athletes because it can increase their risk of suffering premature fatigue during HA and/or maximal exercise [72]. Therefore, improving and optimizing the functionality associated with a health intestinal barrier possibly decreases HS shock and increases tolerance. In this regard, the main stimulus for changing the gut ecosystem and its functionality is diet and hydration. 

From our perspective, hydration should also be emphasized, especially in context where gut dysbiosis combines with heat stress (HS) because dehydration can be an aggravating factor for HA [45]. This leads to a loss of thermoregulatory capacity, further increases core temperature (Tc), and damages the epithelial barrier. A state of dehydration can exacerbate undesired HS responses. In fact, Lambert et al. [45] reported that the loss of fluid balance during running exercise produced greater intestinal permeability than that found in athletes that maintained adequate hydration during the race, even with values similar to those at rest, if losses due to sweat were compensated. Thus, a disbalance of hydration contributes to reduce intestinal blood flow, aggravating intestinal permeability and impairing physical performance [45]. 

It is imperative to emphasize the importance of adequate nutrition to promote GM health and reduce the possibility of induced intestinal dysbiosis [13]. Several studies have investigated how diets rich in saturated fats [73,74] and sugars [75] decrease GM diversity and impair intestinal permeability. In this sense, Pendyala et al. [76] demonstrated how a Western-style diet rich in fats and carbohydrates increased endotoxemia originating in the intestine by 71%, while a diet lower in fats and carbohydrates reduced these levels by 31%. Among sugars, it has been shown that the excessive consumption of fructose is related to impairment of the intestinal barrier [77,78]. While refined sugars promote the growth of opportunistic bacteria such as *Clostridium difficile* [79] and *perfringens* due to increased bile production [80], complex carbohydrates increase levels of beneficial species such as *Bifidobacteria* spp., *B*. *longum* subspecies *longum*, *B*. *breve*, and *B. thetaiotaomicron* [81]. Vegetarian diets prevent the growth of potentially pathogenic bacteria such as *E. coli* and other members of the *Enterobacteriaceae* family [82]. This is due to the greater amount of fiber that this type of diet contains, which causes greater production of short-chain fatty acids (SCFA) by intestinal bacteria, as well as a decrease in intestinal pH [82]. Fiber consumption seems to be another key aspect for maintaining gut eubiosis. In this regard, Filippo et al. [83] reported that the GM of European children is reduced in *Bacteroidetes* and enriched in *Enterobacteriaceae* compared to the GM of African children of rural origin. The main difference between the diet of European and African children is associated with fiber consumption. The consumption of flavonoids of plant origin, such as quercetin present in grapes and onions, which increase epithelial resistance and the expression of claudin-4 in epithelial cells [84], can contribute to the protection against intestinal permeability.

Therefore, above description indicates that nutrition is key to maintaining GM health and avoiding or correcting dysbiosis. Athletes must aim to avoid the excessive regular consumption of sugars and saturated fats, limit the intake of animal proteins, and increase fiber consumption. On the other hand, the stimulus of fasting overnight for 14 to 16 h has demonstrated effectiveness in promoting intestinal homeostasis, reducing inflammation, and regenerating the mucosal barrier [85]. It is recommended to use fasting as a strategy prior to the application of controlled hyperthermic stimuli, to at least maintain a minimum interval of 4 h from the last intake of food before starting the thermal stimulus, whereby intestinal motility is reduced [86]. 

### 3.2. The Effect of Probiotics, Prebiotics, Vitamins, and Short-Chain Fatty Acids on Gut Health to Improve Pre-Acclimatization and Heat Tolerance in Athletes

Here, we propose that increasing beneficial bacteria to maintain good permeability could improve premature heat shock in athletes exposed to heat conditions and during maximal exercise. The intake of probiotics has demonstrated protective benefits in regards to gut health and athletic performance [87]. In this context, it appears that probiotic supplementation may mitigate the adverse effects of heat exposure when implemented for a prolonged duration. Little research exists on the topic; however, Gill et al. [88] demonstrated that supplementation with a probiotic drink containing *Lactobacillus casei* during two hours of running at 34 °C did not significantly improve endotoxemia and elevated cytokine levels caused by HS. However, Shi et al. [56] reported that maintaining probiotic supplementation for 4 weeks and increasing the dose (containing 45 billion CFU of *Lactobacillus, Bifidobacterium,* and *Streptococcus*) could effectively reduce the increased level of LPS caused by athletes exercising at high temperatures. Given the small number of studies on this topic, it is necessary to further explore whether probiotic supplementation can significantly improve HS-related discomfort. Before making recommendations to athletes, it is therefore important to consider: (1) the type and dosage of probiotics, accordingly the individual GM, and (2) the individual factors regarding the host flora status (diversity) and the HS stimuli that the athlete can withstand to better define the intervening role of probiotics [87]. 

Therefore, it may be essential to recommend that athletes test their GM prior to including an HA strategy. The analysis of key bacteria related to gut barrier homeostasis, mucosal production, and regeneration would be important. There are certain protective bacteria which have been accepted as biomarkers of healthy gut barrier permeability (*Akkermansia muciniphyla*, *Faecalibacterium prausnitzii*, *Roseburia*, and some species of *Eubacterium)* [57,58]. Depending on the mucosal related-bacteria proportions, the pre- and probiotic intervention and diet could be individualized and proposed. We have observed that elite endurance athletes expressing high levels of *Faecalibacterium prausnitzii* and *Roseburia* prior to endurance competitions were free of GI complications, even though immediately after exercise, these levels decreased significantly. It seems that prolonged physical effort alters gut microbial activity and functional intestinal activity, presenting as gut permeability, but the reduction of food intake may also be related to gastrointestinal motility and endocrine metabolites, such as leptin, ghrelin, and neurotransmitters. Nutritional and probiotic administration prior to maximal physiological effort could be an interesting strategy to minimize the possible negative effects of stimulus such as HA, hypoxia, and/or prolonged maximal exercise on gut microbiota. The administration of some probiotics suggested in Refs. [89,90], as well as others, such as *Escherichia coli Nissle 1917* (EcN), has been demonstrated to be effective in preventing the barrier disruption caused by infection of T84 and Caco-2 cells with an enteropathogenic *Escherichia coli* strain [91]. 

Another probiotic strain, *Lactobacillus plantarum MB452* (from the VSL3 probiotic), induces the transcription of genes like occludin and cingulin that improve gut barrier integrity [92]. A study has shown that *L. plantarum* can regulate human epithelial TJ proteins in vivo and confer protective effects against chemically induced epithelial barrier disruption in an in vitro model [93]. Administration of *L. plantarum* in the duodenum of healthy human volunteers significantly increases zonuline and occludin near the TJ structures [93]. These findings suggest that *L. plantarum* administration may enhance the stability of TJ complexes in humans and attenuate their disruption by cytokines, toxins, and pathogens. Besides *L. plantarum*, other *Lactobacillus* probiotic strains also show protective effects on the intestinal barrier. Among these strains are *L. salivarius UCC118*, *L. salivarius CCUG38008*, *L. rhamnosus GG*, the *Lactobacillus casei DN-114.001* strain, and the *Lactobacillus casei Shirota* strain [94,95,96,97,98].

Taking certain vitamins has also proven to be effective in improving GM balance. Vitamin A and its derivatives have been shown to regulate the growth and differentiation of intestinal cells [99]. Vitamin D also appears to play a role in intestinal barrier. Vitamin D deficiency, a characteristic of inflammatory bowel disease (IBD), correlates with certain disease severity [100]. Supplementation with ascorbic acid can naturally reduce post-exercise LPS concentration by ~12 fold [101]. Supplementation with glutamine in athletes with inadequate levels can improve intestinal barrier health, as previously described in response to extreme stressors, including altitude shock and heat shock in humans [102]. Short-chain fatty acids (SCFA), including butyrate, play a particular role in maintaining the intestinal barrier [103]. To elevate SCFA metabolites, it is important include prebiotics in the diet daily, especially galactooligosaccharides (GOS) [104] and fructooligosaccharides (FOS) [105], as well as resistant starch that feeds SCFA-producing bacteria. Among these, butyrate stands out for its role in maintaining the intestinal barrier [103]. According to a study using rats with colitis induced by dextran sodium sulfate [106], the administration of butyrate could be useful for a recovery of transepithelial resistance associated with the maintenance of the integrity of tight junctions and the inhibition of the release of inflammatory factors such as TNFα.

Regarding supplementation, it is crucial to meticulously investigate the benefits of its use because, as suggested Álvarez-Herms et al. [30], a possible association between supplement intake and deterioration of the gut microbiota (GM) could appear. Supplements and probiotics include normal industrial additives that, in excess, may exacerbate reactive intestinal responses, increasing gut bacterial dysbiosis. Food additives present in functional nutritional products, such as probiotics and/or supplements, are numerous, including preservatives (sulfur dioxide, sodium sulfide and/or benzoate, etc.), flavor enhancers (monosodium glutamate), artificial sweeteners (sucralose, xylitol, sorbitol, saccharin, etc.), colorants (titanium dioxide), and emulsifiers [30,107]. Therefore, it is important to consciously evaluate the quality and quantity of the supplementation of athletes, specifically considering the precise nutritional of athletes in regards to individual GM balance.

**Figure 3:**
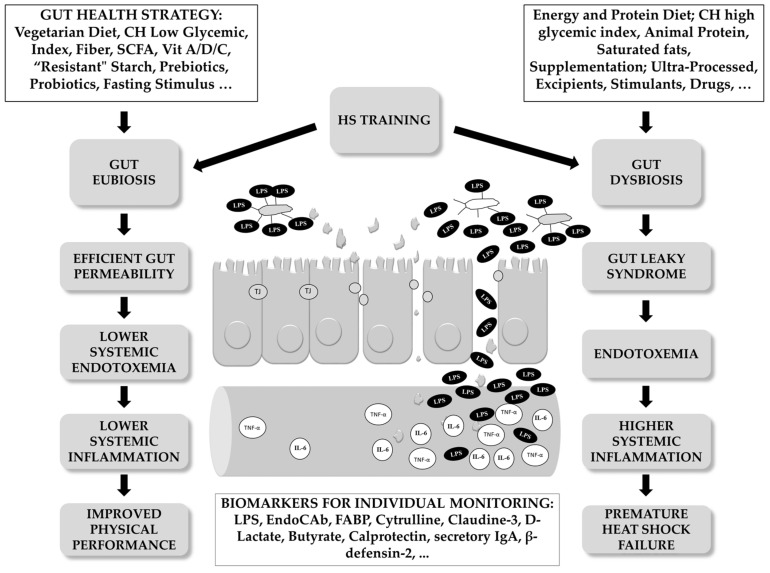
Heat stress training in athletes can lead to positive adaptive physiological responses or contrastingly, promote aberrant physiological consequences. HS can promote or aggravate GM dysbiosis. Athletes are required to maintain systemic health to reach their maximal biological potential and acquire physiological benefits from the supported stimulus. Athletes with poor consideration of their own health are exposed to GM dysbiosis and tolerate poor maximal physical exercise and another stimuli, including HS or hypoxia.

As can be seen in Figure 3, different strategies can promote GM eubiosis and improve both systemic health and physiological performance. Contrarily, the maintenance of chronic daily habits that negatively affect GM homeostasis may exacerbate inflammatory responses, aggravating intolerance to HS and physical exercise.

Approaches for preserving gut health and improving the adaptation to HS stimuli can focus on both enhancing the consumption of certain foods and substances beneficial to gut eubiosis and avoiding those that may disrupt gut balance and heat tolerance. In this regard, it has been reported that the intake of certain medications, such as anticholinesterases, antidepressants, diuretics [108,109], or non-steroidal anti-inflammatory drugs, can lead to disturbances in sweating, thermoregulation [110], intestinal inflammation [111], and can eventually contribute to the exacerbation of gut dysbiosis and leaky gut syndrome. Alcohol intake has also been linked to increased intestinal permeability and consequent endotoxemia [94,112].

## 4. Gut Health as the Key Factor to Individualize Stimulus of Physical Exercise and HA

Before recommending that athletes follow specific training plans, including environmental stimulus such as hyperthermia and/or hypoxia, it is necessary to take into consideration the GM balance to promote positive interventions. Monitoring individual pre- and post-GM changes after different HA interventions appears to be a key strategy to enhance the individualization of an athlete’s systemic health and favor greater adaptability [111]. Different markers to determine the state of the GM and gut permeability have been reported, such as (see Figure 3): (1) levels of systemic LPS [22,23,39], (2) circulating antibodies against the endotoxin core (EndoCAb) [113,114], (3) fatty acid binding proteins (FABPs) [115,116,117], (4) fecal calprotectin [113], (5) secretory IgA [115], and (6) fecal β-defensin-2 [117].

In summary, there is limited evidence regarding the effects that a healthy GM can produce on HA tolerance and the consequences associated with heat reactive mechanisms. Nevertheless, it is imperative to investigate the precise mechanisms underlying functional communications between the microbiota and physiological HS responses to timely adjust nutrition, hydration, and probiotic levels. Finally, we have seen that despite the evident health and performance benefits that exposure to heat during HA can offer to athletes, this practice can sometimes induce aberrant systemic alterations, such as the deterioration of GM functionality, manifesting as dysbiosis and increased intestinal permeability. At times, certain controlled heat stimuli do not induce positive adaptive responses because athletes suffer from dysbiosis that predisposes and exacerbates these alterations.

Understanding individual susceptibility to HA is essential for optimizing both performance and health [118]. From a practical perspective, athletes include HA training, with poor control of their own individual levels of tolerance, where stimulus could be optimal to improve physiological responses. Therefore, before including HA in a training regimen for athletes, these athletes should test their individual threshold of tolerance at different systemic levels, including metabolism, osmolarity, and GM changes, if was possible.

In athletes, the state of the GM is crucial, as it enhances physical performance and overall health. The GM also plays a role in regulating intestinal permeability and the inflammatory response in response to HS. However, further research is required to fully understand the relationship between HA, the GM adaptive responses, and specific probiotic/nutritional interventions. Studying the physiological, genetic, biochemical, behavioral, and molecular variables may be key to developing personalized training strategies in athletes. Omics sciences could play a fundamental role in this approach.
